# Determinants of Bangladeshi patients' decision-making process and satisfaction toward medical tourism in India

**DOI:** 10.3389/fpubh.2023.1137929

**Published:** 2023-05-02

**Authors:** Muhammad Zakaria, Muhammad Aminul Islam, Md Khadimul Islam, Aklima Begum, Nahida Akter Poly, Feng Cheng, Junfang Xu

**Affiliations:** ^1^Department of Communication and Journalism, University of Chittagong, Chattogram, Bangladesh; ^2^Department of Communication, Wayne State University, Detroit, MI, United States; ^3^Department of English, Shaikh Burhanuddin Post Graduate College, Dhaka, Bangladesh; ^4^Department of English, Daffodil International University, Dhaka, Bangladesh; ^5^Vanke School of Public Health, Tsinghua University, Beijing, China; ^6^Institute for Healthy China, Tsinghua University, Beijing, China; ^7^Center for Health Policy Studies, School of Public Health, Zhejiang University School of Medicine, Hangzhou, China; ^8^Department of Pharmacy, Second Affiliated Hospital, Zhejiang University School of Medicine, Hangzhou, China

**Keywords:** medical tourism, tourism destination, medical tourism costs, country environment, facility, services

## Abstract

**Objectives:**

The aims are to explore the factors influencing Bangladeshi patients' decision-making process and their satisfaction level toward medical tourism in India.

**Methods:**

The study used a quantitative research approach with a cross-sectional survey. Data were collected from the patients or their relatives (*N* = 388) who would have decided to travel to India for medical and treatment purposes at the Chittagong Indian visa center (IVAC). Data were collected using a structured, pre-tested, and facilitator-administered questionnaire, which mainly included the social demographic characteristics, health status, medical tourism information and medical tourism index. Hierarchical regression analysis was performed to explore the factors influencing their satisfaction level toward medical tourism in India.

**Results:**

More than three-fourths of the participants had visited India for self-treatment. Of the participants, 14% were cardiology patients, and 13% suffered from cancer. The relatives were the key source of information regarding medical tourism for more than one-fourth of the respondents. India's availability of well-experienced doctors, hospital/medical facilities with high standards, well-trained doctors, reputable doctors, and quality treatments and medical materials were top-ranked items. Regression results depict that facility and services appeared as the strongest factor (β = 0.24, *t* = 4.71, *p* < 0.001) followed by tourism destination factor (β = 0.16, *t* = 3.11, *p* = 0.002), medical tourism costs factor (β = 0.16, *t* = 3.24, *p* = 0.001) and country environment factor (β = 0.15, *t* = 2.69, *p* = 0.007).

**Conclusions:**

We found that the factor related to facility and services is one of the strongest predictors in our models. Therefore, home countries must strengthen the health care providers' advanced professional training, including service attitudes. Moreover, it is important to lessen the language barrier, reduce the airfare for medical tourists, and make the treatment cost more affordable for patients.

## Introduction

Medical tourism is a phenomenon that has attracted much interest due to not only its substantial economic impact but also the changing landscape of global healthcare delivery services (1–4). As a new form of tourism and industry, medical tourism has been one of the fastest-growing tourism sectors in recent years (5–9).

With increasing medical costs, long waiting lists, and lack of availability of some treatments, combined with the easiness of global travel and the fast advancements in technology, medical tourism has become more alluring to patients worldwide (10–12). People who need to receive some important medical treatments such as cardiac, orthopedic, dental, and plastic surgeries are going to crucial destination countries famous for overseas patients (e.g., India, Singapore, and Thailand) (13, 14). In recent years, more people have been traveling to foreign countries for cost-effective medical treatments, which are often packaged with complementary tourism services such as hotel accommodation or sightseeing (12, 13). Correspondingly, medical tourism has experienced rapid growth in recent years. Approximately 14 million cross-border patients travel worldwide every year, spending an average of $3,800–7,000 per visit on medically-related costs, transnational and local transport, inpatient stay, and accommodations (15). Within the United States alone, approximately 1,400,000 Americans traveled outside the country for medical purposes in 2017, and over 40% of Americans consider medical tourism a practical future option for themselves (16). It is reported that Thailand, Singapore, and India have the highest share of the medical tourism market in Asia (8). In 2008, Thailand generated USD 1.5 billion from its medical tourism industry (2).

India, one of the global leaders in the medical tourism industry, has emerged as one of the most cost-efficient and fastest-growing medical tourism destinations today for patients worldwide (17–21). The uniqueness of India is its ability to offer holistic medical services such as Unani, yoga, meditation, Ayurveda, and homeopathic treatments (22). In 2012, the medical tourism revenue of India represented 14 percent of total tourism revenues or 1.8 percent of the world's GDP, and India alone provided $2.3 billion in medical services to foreign tourists during this period (23).

Nowadays, an increasing number of Bangladeshi people are seeking medical treatment abroad (24). According to the media report (24), Bangladeshi people go abroad for medical purposes due to the high cost of surgery, uninsured, long waiting periods, non-availability of treatment, lack of medical facilities and proper care, shortage of trained doctors and nurses, ethical and regulatory reasons, corruption, and inadequate public or private medical facilities in the country. In addition, the behavioral problem of doctors is one of the main reasons for the flock of patients to India. Here, a common allegation about the doctors in Bangladesh is not being attentive to the patients. Most of the specialized doctors in the capital city even do not give 5 min to a patient. Due to the failings of the legal system of Bangladesh, doctors and hospitals do not fear the consequences of their negligence, resulting in the suffering of the patients instead of the treatment. Even though political leaders and even doctors go abroad for medical treatment, which causes people to lose confidence in the doctors. Besides, long waiting times for top-rated and specialized doctors also resulted in flocking patients to India for treatment. Furthermore, without a hygienic environment at tourist destinations, hospitals, hotels, and restaurants, people from distant areas are reluctant to come to Dhaka, the capital city, despite having a number of outstanding doctors.

According to the reports of various media and research articles, more than 300,000 people from Bangladesh go abroad for medical treatment every year, mainly to India. Notably, the majority of overseas consumers of Indian healthcare services are from Bangladesh and stands at 220,000 in the year 2017 (18). It is estimated that more than 165,000 of the 460,000 foreign patients that entered Indian hospitals are from Bangladesh, making up one in three (25). Nonetheless, very few studies were conducted among Bangladeshi tourists who traveled to India for medical purposes. So, doing more scholarly work to add documentation in this regard is rational. Under this background, we aim to explore the factors regarding the decision-making process of medical tourism and satisfaction among Bangladeshi patients, which may help policymakers adjust related policies for improving residents' health by further meeting their medical needs. The paper begins with a review of existing medical tourism literature, followed by the theory of planned behavior as the theoretical framework. The following section introduces the study methods and results. Furthermore, the discussion section emphasizes a summary of the study and the theoretical and practical implications.

## Literature review

### Concept of medical tourism

Medical tourism was initially attributed to travel firmly for medical intervention, and many authors were more likely to define it as consonant with this view (12). For instance, Hume and DeMicco (26) implied medical tourism as “the process of traveling to another country to receive medical, dental, and surgical care” (p. 76). Likewise, Sobo (27) defines it as “contemporary travel for the primary purpose of obtaining indicated or elective dental or biomedical services” (p. 326).

Some definitions emphasize the intention of the patient and their relatives. Johnston et al. (28) prefer “patients leaving their country of residence outside of established cross-border care arrangements made with the intent of accessing medical care, often surgery, abroad” (p. 1). Similarly, Lunt and Carrera (29) confine the definition of medical tourists to “patients who are mobile through their own volition” (p. 28).

For this research, a more holistic definition is used. Therefore, throughout the study, medical tourism refers to a person's traveling to both developed and developing countries with the aim of invasive or elective medical treatments ranging from invasive surgery to health check-ups driven being holidaymakers with direct or indirect involvement in leisure, relaxation, and business coordination with hospitality and tourism industry.

### Health and wellness tourism

For the last two decades, the term “medical tourism” has been used recurrently to comprise both medical and health and wellness tourism, partly due to the blurring of the lines between medical treatments and health enhancement (12). Accordingly, Bookman and Bookman (30) preferred to refer to the entire business as “medical tourism,” claiming that this represents the expanding meddling of medicine, even in spa and wellness services (p. 43). Therefore, Hudson and Li (12) concentrated on the wellness of health as they defined it as travel (domestic or international) for the primary goal of invasive, diagnostic, or lifestyle medical therapies (p. 230).

Heung et al. (6) described it as a holiday that entails crossing foreign borders in order to receive a wide range of medical treatments. Activities for enjoyment, relaxation, and leisure are frequently included, along with services for wellness and healthcare. Similar viewpoints have the capacity of pursuits in which a person frequently travels large distances or crosses international borders to access medical care while actively or passively engaging in leisure, business, or other purposes (31). Such treatment is becoming more closely linked with tourist activities to help international patients adjust to a new cultural setting and keep them occupied during the pre-and post-operative periods (32). Connell (5) describes medical tourism as an industry where people frequently travel great distances to foreign nations to receive medical, dental, and surgical care while also vacationing in a more traditional sense.

### Collaboration with the hospitality industry

A growing body of literature claims that a medical tourism product may be a medical service with a leisure component (5, 6, 31–33). Although coordinating the assets and services of the tourism sectors and health service is a challenge, Heung et al. (6) claim that strategically such coordination is often carried out at the governmental level. Once an individual intends to have a medical procedure performed in the destination country, they need both healthcare services and tourism facilities. Comprehensive travel arrangements, including obtaining visas, and airline tickets, must be made, the availability of a doctor must be ascertained, and other medical provisions, including recuperation services, must be planned (6). All of these services require cooperation as well as coordination between the two sectors (34). As the acceptance and reputation of medical tourism continue to grow, there are enormous opportunities for both the hospitality and healthcare industries (26). For example, a patient could spend an extended period recuperating in a luxury hotel after surgery. The hotel would need to offer together with specially designed rooms and proper follow-up care in coordination with a medical expert. Safety and care are necessary where health and medical services are concerned and for those traveling to other countries to get such services. Therefore, a well-coordinated partnership between medical service providers and hotels is required to meet medical tourists' requirements (6).

### Key variables of medical tourism

A growing number of studies explored different factors associated with various aspects of medical tourism, for example, selecting the destination country for medical tourism, engaging in medical tourism, etc. Studies exploring medical tourism mention the low cost of treatment and check-ups, limitations of insurance coverage, availability of medical procedures for complex treatment and surgery, capacity to obtain treatment more quickly, privacy issues, employer and insurance company endorsements, and higher quality care and medical service providers as factors determining the decision to seek overseas medical care (5–7, 9, 17). Cost and quality are considered essential elements of the medical tourism sector (18, 21, 35). Affordability and service quality of hospitality and tourism have also been considered key features for a medical tourism destination (9, 36, 37).

Further, Zolfagharian et al. (17) explore that domestic medical costs, patient privacy concerns, medical restrictions, and foreign destination desirability influence medical tourism considerations. Moreover, Österle et al. (38) suggest that the cost, accessibility, and quality of the treatment primarily influence a patient's decision regarding the destination. Other factors, such as the care destination's culture, social factors, and institutional environment, come in second. Literature also highlights the value of credibility, perception, and the need for accreditation, certification, and international standards (39, 40). Ebrahim and Ganguli (14) and Beladi et al. (15) have investigated the role of human resource development and administrative efficiency.

According to studies by Bagga et al. (18), Cham et al. (40), and Olya and Nia (41), nation-specific characteristics and features like country knowledge, culture, language, accessibility, safety, and security have also been examined. By researching the appeal of a medical tourism destination, tourism-specific aspects of the destination nation, such as weather, attractions, culture, and exoticness, have also been taken into account (36, 42). Conducting an empirical study on sub-Saharan African medical travelers, Khan et al. (43) suggest that perceived risks and travel constraints of medical travelers negatively affect their perception of the different aspects of medical tourism destinations and impact their travel behavior. Likewise, Chelliah et al. (44) found that perceived destination image was reported as a significant factor of visit intention among medical tourists.

### Conceptual model of medical tourism

During the last few decades, several models have been developed to identify factors affecting different aspects of medical tourism, such as the destination country, hospital selection process, and other decision-making processes. Han and Hyun (45) proposed a model clarifying the intentions of medical tourism by considering the impacts of quality, trust, satisfaction, and affordable pricing in the destination country (45). A two-stage model was proposed by Smith and Forgione (46), suggesting the factors influencing a patient's choice to travel abroad for medical treatment. Their model indicates that in the first stage, country-specific characteristics, such as economic conditions, political climate, and regulatory policies, have an impact on the selection process of the destination country, whereas treatment and service costs, hospital accreditation, quality of services, and physician training affect the choice of healthcare facilities (46).

In order to develop a conceptual framework for the decision-making process of medical tourists based on different models used in tourism literature, Khan et al. (47, 48) argue that risks associated with medical tourism, motivations of medical tourism, and linkage of destination's image with risks and motivations are associated with medical tourists' travel process.

Despite the copious erudite attempts to comprehend and elucidate the concept, nature, and different facets of medical tourism and to explore the associated factors, this body of study attempts to add to the literature by exposing the primary reasons, means, and decision-making process of the patients for traveling from a developing country to another developing country along with the exploration of the factors promoting the medical tourism. In addition to this, there is a dearth of scholarly works that addressed Bangladeshi medical tourists. This study is an attempt to fill up this gap.

### Theoretical framework

The theory of planned behavior is one of the most widely used psychological models investigating the factors affecting behavior (49, 50). It enables the detection of significant behavioral effects that can be used to forecast and modify behavior. According to the hypothesis, if a person controls their behavior, behavioral intention can most closely predict that behavior. Moreover, attitudes toward the action, perceptions of significant others' support of engaging in the behavior (subjective norms), and perceived control over engaging in the behavior all predict behavioral intention. The way someone feels about an action is referred to as their attitude. A person might, for instance, feel either favorably or unfavorably about visiting any nation for medical treatment. The term “subjective norm” describes how an individual thinks about whether or not important others find their behavior to be acceptable. For instance, if an individual believes their family and friends approve of it, they may pursue medical tourism. The ability or capacity of the person to successfully carry out the activity is referred to as control over the behavior. Language and cultural obstacles, the capacity for long flights, and the courage to face challenges while ill and recuperating in a foreign country are all perceived control factors that could operate as barriers to successful medical tourism participation (50). Hence, according to the theory of planned behavior, a person's attitude, subjective norm, and sense of control over their behavior all influence their intention (49, 50). To the best of the researchers' knowledge, several earlier studies used the planned behavior theory to forecast medical tourism preferences (7, 51–53). Accordingly, we also suggested the theory of planned behavior as a suitable framework for the current study.

## Methods

### Study design and sample size

We designed quantitative research with a cross-sectional questionnaire survey among the patients or their relatives who would have decided to travel to India for medical purposes.

The patients or their relatives who would have decided to travel to India for medical and treatment purposes were the study population. The study was conducted from January 15, 2022 to March 20, 2022 in a limited period due to an adequate budget. Survey respondents were selected using convenience sampling rather than random sampling since a cohort of the respondents were patients, and they preferred to participate voluntarily, given their health status. Convenience sampling was also recommended by Manaf (54) for any studies on patients, and some previous studies used it for sample selection (7, 11, 51, 53, 55).

The sample size was determined using single population proportion formula considering the following assumption: probability (p) = 50% (because we did not have information on any previous study conducted in Bangladesh on the same research topic, to begin with, we assumed that half of the population might be satisfied toward medical tourism in India), significance level 5% (α = 0.05), Z $\frac{\alpha }{2}$ = 1.96 and margin of error 5% (d = 0.05).


$$\begin{array}{l} \text{n }=\;\frac{{\left(\text{Z}\frac{\alpha }{2}\right)}^{2}\text{p}\left(1-\text{p}\right)}{{\text{d}}^{2}}\\ \;=\;\frac{1.96{\;}^{2}\times 0.50\;\times \;0.50}{0.05^{2}}\;\\ \text{n }=\;384 \end{array}$$

However, we invited 400 patients to participate in the study and among them, 388 filled up the questionnaires with a response rate of 97%.

### Questionnaire design

The data were collected through a facilitator-administered and structured questionnaire survey. The questionnaire was prepared based on reviewing related literature, and the factors influencing the Bangladeshi patients' decision-making process regarding medical tourism in India were adapted from a previous study (36). The questionnaire was divided into four sections, i.e., (a) patients' sociodemographic and socioeconomic characteristics, for example, education, gender, socioeconomic status, area of residence, and marital status; (b) health-related background information, such as perception of health status, health insurance coverage, the purpose of visiting India, duration of disease before visiting India, sector of receiving treatment in Bangladesh; (c) source-related information, for instance, a key source of information regarding medical tourism, an influential source for decision-making; (d) influencing items of the Bangladeshi patients' decision-making process regarding medical tourism in India. The questions were finalized after a pilot study was carried out with 40 participants to ensure the quality of the questionnaire.

### Measurements

The determinants influencing the Bangladeshi patients' decision-making process regarding medical tourism in India were the predictor variables of this study. It was assessed using the ‘The Medical Tourism Index (MTI)' which consists of 34 items developed by Fetscherin and Stephano (36). Six-point Likert scale was used to measure 34 items related to medical tourism (MTI) as: Do not know = 0, strongly disagree =1, disagree = 2, uncertain = 3, agree = 4, strongly agree = 5.

### Data collection

The questionnaire survey was conducted by 10 data collection facilitators who were recruited based on their previous experiences in data collection. At first, they approached and requested the visitors to participate in the survey who came to apply or take the medical visa at the Chittagong Indian visa center (IVAC) during the study period. At that time, data collection facilitators explained the importance and purpose of the study to visitors while they were waiting to get their medical visas or leaving the premises after submitting the visa application. After obtaining the consent, the confidentiality of the study was addressed to them, and upon their agreement on voluntary survey participation; data were collected through a face-to-face questionnaire survey. After completing the survey, facilitators requested the study participants to give their contact numbers for collecting follow-up information about their satisfaction with medical tourism in India. The study participants were assured that their contact numbers would be used only for research purposes and be maintained confidentiality. After a period of time, the data collection facilitator contacted the patients who traveled to India for medical and treatment purposes and asked the follow-up question about the satisfaction level toward the medical tourism in India.

### Content validity and reliability

In the first stage items of the MTI were translated from English text into Bengali (interview data) as the original scale was in English and the target study participants were the Bengali patient. A translation of the MTI into Bangla was completed by two translators (one was a psychologist and the other was the author of this manuscript). They were native Bangla speakers knowledgeable of assessment principles and in-depth knowledge of their native Bangla language. They evaluated the meaning of the equivalence of items. The expert panel next evaluated the scale's forward translation to see whether there were any variations between the two language versions. Two linguists conducted a back translation of the scale (translating things from Bangla to the original English) (one was a linguist and the other was a faculty in English). The expert panel also evaluated the scale's back-translated version, concluding that it was a suitable translation of the original English scale. The expert group evaluated the two translations before completing the MTI-B. The original English scale was compared to the MTI-B after that. The first round of the original MTI was given to 40 responders who were sufficiently fluent in both Bangla and English. The same 40 participants who took part in the pilot research previously were given the MTI-B a few days later using the original English scale. The MTI-B was a suitable translation of the original MTI, according to a substantial positive correlation (r = 0.928, p 0.01) between the two versions of the scale. Besides, the reliability of the scale was also checked. McDonald's omega (ω = 0.92) and Cronbach's alpha (α = 0.92) values suggested very good internal consistency of the scale.

### Statistical analysis

The data were coded and entered into IBM SPSS version 24.0. To estimate the proportion of variance in study participants' satisfaction with medical tourism in India that can be accounted for by MTI-related factors, a hierarchical linear regression analysis was performed. All four sub-factors were included in linear regression models to determine the predictors of better satisfaction scores as their *p-*value was < 0.05. In hierarchical regression, step 1 assessed the determinants of a more satisfying level by country environment factor. Step 2 explored the effects of country environment factors and tourism destination-related factors. Step 3 examined the influence of the country environment factor, tourism destination factor, and medical tourism costs-related factor while step 4 (final model) investigated the country environment factor, tourism destination factor, and medical tourism costs and facility and services-related factors. In the model summary (**Table 4**), ANOVA values (*p* < 0.001) of each step for the satisfaction level report that our hierarchical regression model performed well and was a good predictor of the main outcome variables. *R*^2^ of each step was changed considerably, and F changes were also statistically significant (*p* < 0.001). Variables having a *p*-value < 0.05 in the regression analysis were taken as significant predictors.

## Results

### Characteristics of study participants

Table 1 shows the sociodemographic characteristics of the study participants. A total of 388 persons participated in the study. Of them, 68 (17.5%) attained up to HSC, while 128 (33%) completed Honors/Bachelor and 192 (49.5%) had an education of Masters. Among the study participants, 104 (26.80%) were female, whereas 284 (73.20%) were male.

**Table 1:** Sociodemographic characteristics of study participants (*N* = 388).

**Variables**	**Categories**	**Frequency**	**Percentage**
**Education**	Up to HSC	68	17.5
	Honors/Bachelor	128	33.0
	Masters	192	49.5
**Gender**	Female	104	26.80
	Male	284	73.20
**Age (M 38.28 years, SD 11.88)**	≤ 30 years	116	29.9
	30–40 years	140	36.1
	>40 years	132	34.0
**Income group**	Lower	96	24.7
	Middle/upper	292	75.3
**Area of residence**	Sub-urban/Rural	84	21.6
	City	304	78.4
**Marital status**	Single/divorced	104	26.8
	Married	284	73.2
**Occupation**	Housewife	52	13.4
	Student	92	23.7
	Business	76	19.6
	Service/Professional	168	43.3

HSC, Higher Secondary Certificate.

Among the study participants, 96 (24.7%) were from lower income group, whereas 292 (75.3%) belong to the middle/upper income group. The majority of the respondents (304, 78.4%) were from city areas while 84 (21.6%) were from suburban/rural areas.

Of the participants, 284 (73.2%) were married, while 104 (26.8%) were single/divorced. Table 1 also illustrates the professional status of the study participants reporting that 168 (43.3%) were service holders/professionals followed by students/others (92, 23.7%), businessmen (76, 19.6%), and housewife (52, 13.4%).

### Health-related characteristics

Table 2 indicates that 176 (45.4%) had a perception of fair health, 140 (36.1%) traveled to India before going for medical tourism purposes, and more than three-fourths of the participants (296, 76.3%) visited India for self-treatment. Among the participants, 120 (30.9%) suffered from the disease for < 1 year before going to India, and 308 (79.4%) received treatment in a private clinic before going to India.

**Table 2:** Health-related characteristics of study participants.

**Variables**	**Categories**	**Frequency**	**Percentage**
Perception of health status	Good	152	39.2
	Fair	176	45.4
	Poor	60	15.5
Specific purpose of visiting India	Self-treatment	296	76.30
	Diagnosis/check-up	92	23.70
Duration of suffering a disease	< 1 year	116	29.9
	1–2 years	116	29.9
	3–4 years	44	11.3
	>4 years	108	27.8
Sector of receiving treatment in Bangladesh	Private clinic	308	79.4
	Government hospital & others	80	20.6

### The diseases that participants intended to be treated during medical tourism

**Figure 1:**
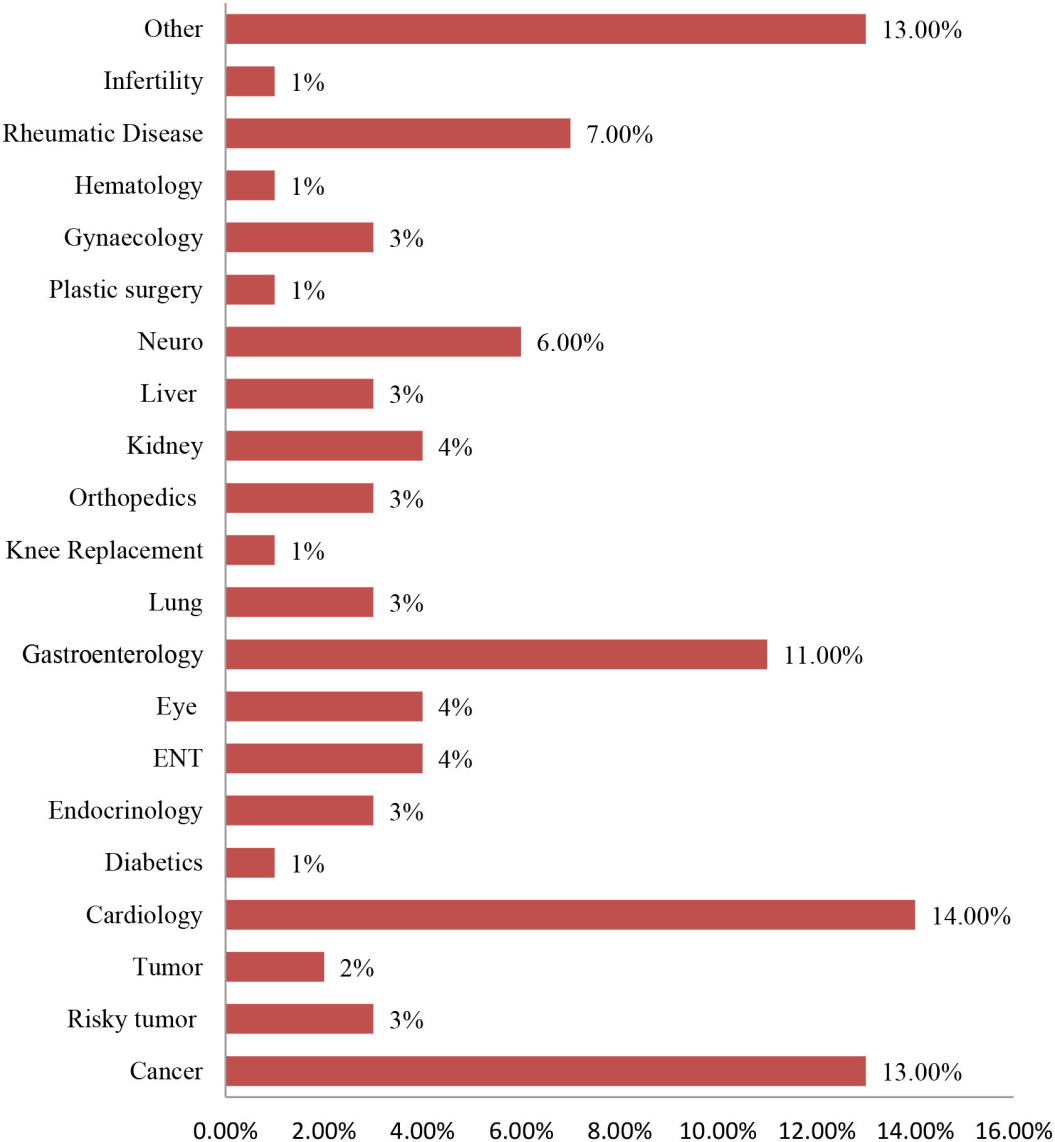
The diseases for study participants intended to take treatment in India.

Figure 1 reports that of the study participants, 14% were cardiology patients, followed by cancer (13%), other diseases (13%), gastroenterology (11%), rheumatic disease (7%), neuro (6%), kidney (4%), eye (4%), ENT (4%), gynecology (3%), liver (3%), orthopedics (3%), lung (3%), endocrinology (3%), risky tumor (3%), tumor (2%), infertility (1%), hematology (1%), plastic surgery (1%), knee replacement (1%), and diabetes (1%).

### Source of information and decision-making process

**Figure 2:**
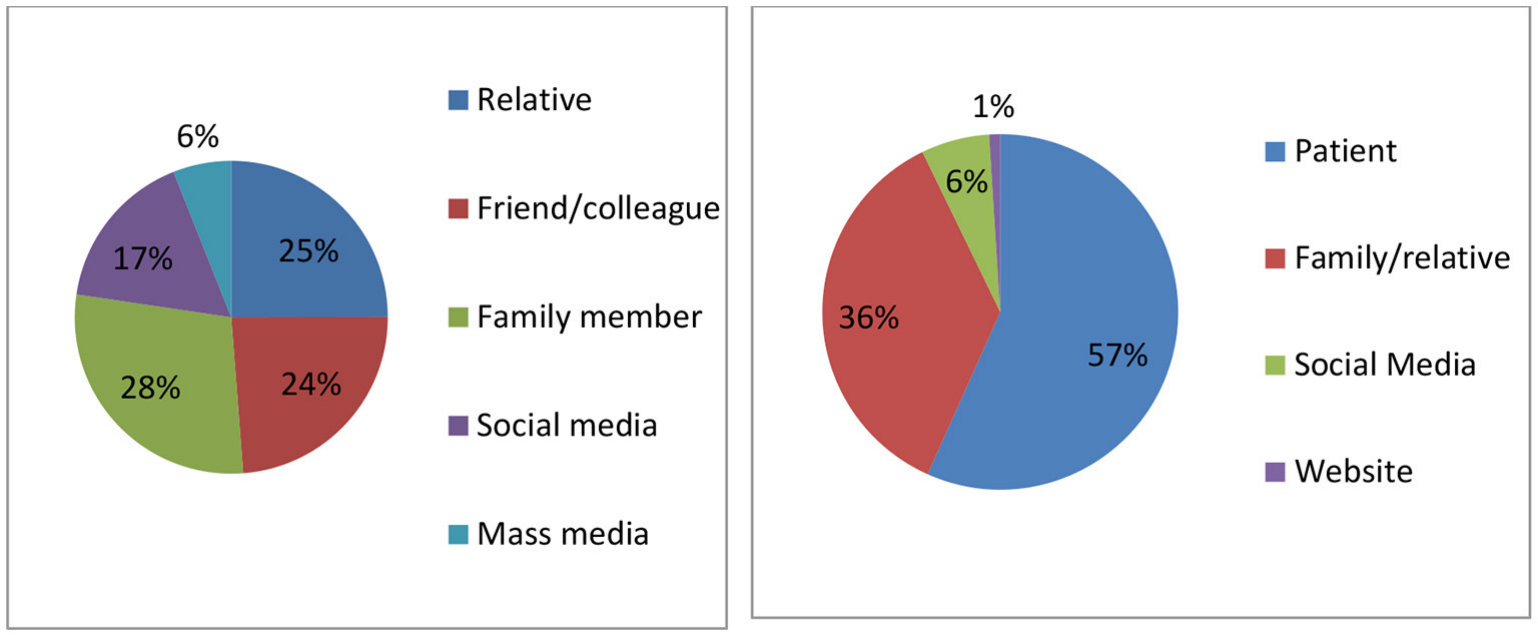
Distribution of the study participants' key source of information of medical tourism in India **(left)** and influential source for decision-making **(right)**.

Figure 2 depicts that interpersonal channels are more prevalent as the source of information about medical tourism in India than media channels. For example, a family member was the key source of information regarding medical tourism for more than one-fourth of the respondents (28%), followed by relatives (25%) and friends/colleagues (24%). Furthermore, 57% of patients decided by themselves to travel to India for the treatment, followed by family members/relatives (36%).

### Distribution of the items related to the medical tourism index

Percentage distributions and mean with one-sample *t*-test (two-tailed) of respondents' attitudes toward the items related to country environment factor were reported in Table 3. The findings from the one-sample *t*-test depict that out of 34 MTI-related items, the mean scores for 20 items were highly satisfactory (*p* < 0.001), while those for 11 statements were below the satisfactory level (*p* < 0.001).

**Table 3:** Mean score and one-sample *t-*test of different items relating to medical tourism index.

**Items**	**M**	**SD**	**Rank**	***t***	***p***
Item 1. India is culturally similar to mine	3.27	1.18	27	−12.20	< 0.001
Item 2. India has low corruption	3.47	1.45	25	−7.13	< 0.001
Item 3. India has a similar language to mine	2.99	1.24	30	−16.04	< 0.001
Item 4. India has a stable economy	2.97	1.63	31	−12.46	< 0.001
Item 5. India is safe to travel to	3.79	1.19	23	−3.40	0.001
Item 6. India has overall a positive country image	3.91	1.18	21	−1.55	0.121
Item 7. India has a stable exchange rate	3.07	1.54	29	−11.84	< 0.001
Item 8. India is an attractive tourist destination	4.28	1.06	16	5.15	< 0.001
Item 9. India is a popular tourist destination	4.21	1.12	18	3.62	< 0.001
Item 10. India has many cultural or natural attractions/sites	4.52	0.68	8	15.02	< 0.001
Item 11. India is an exotic tourist destination	4.30	1.20	15	4.89	< 0.001
Item 12. India has great weather	3.20	1.30	28	−12.19	< 0.001
Item 13. India is low cost to travel to	3.86	1.10	22	−2.58	0.010
Item 14. India has low accommodation costs	3.60	1.19	24	−6.65	< 0.001
Item 15. India has low treatment costs	3.94	1.14	20	−1.07	0.286
Item 16. India has affordable airfares to travel to	3.34	1.32	26	−9.87	< 0.001
Item 17. India has low healthcare costs	3.86	1.20	22	−2.36	0.019
Item 18. India has quality treatments and medical materials	4.60	0.68	5	17.21	< 0.001
Item 19. India has hospital/medical facilities with high standards	4.66	0.52	2	25.18	< 0.001
Item 20. India has well experienced doctors	4.74	0.46	1	31.72	< 0.001
Item 21. India has well–trained doctors	4.65	0.72	3	17.78	< 0.001
Item 22. India has reputable doctors	4.64	0.54	4	23.25	< 0.001
Item 23. India has internationally certified staff and doctors	4.26	1.27	17	3.99	< 0.001
Item 24. India has hospital/medical facilities with good healthcare indicators	4.43	0.97	12	8.76	< 0.001
Item 25. India has doctors I would recommend to my family or friends	4.44	1.00	11	8.77	< 0.001
Item 26. India has reputable hospitals/medical facilities	4.60	0.57	5	20.70	< 0.001
Item 27. India has friendly staff and doctors	4.54	0.83	6	12.77	< 0.001
Item 28. India has overall a positive medical tourism image	4.51	0.75	9	13.29	< 0.001
Item 29. India is known for state–of–the–art medical equipment	4.12	1.34	19	1.83	0.069
Item 30. India has internationally accredited hospitals/medical facilities	4.32	1.16	14	5.41	< 0.001
Item 31. India has internationally educated doctors	4.32	1.29	14	4.88	< 0.001
Item 32. India has hospitals/medical facilities I would recommend	4.53	0.84	7	12.20	< 0.001
Item 33. India has high quality in healthcare	4.46	0.93	10	7.13	< 0.001
Item 34. India has internationally certified doctors	4.34	1.29	13	16.04	< 0.001

Test cut off value: 4.

Based on the mean value, as it is observed, among 34 items most of the top ranked items were from the facility and services (FS) factor. According to the rank order, the top 15 items were: (a) India has well-experienced doctors (FS); (b) India has hospital/medical facilities with high standards (FS); (c) India has well-trained doctors (FS); (d) India has reputable doctors (FS); (e) India has quality treatments and medical materials (FS); (f) India has friendly staff and doctors (FS); (g) India has hospitals/medical facilities I would recommend (FS); (h) India has many cultural or natural attractions/sites (TD); (i) India has overall a positive medical tourism image (FS); (j) India has high quality in healthcare (FS); (k) India has doctors I would recommend to my family or friends (FS); (l) India has hospital/medical facilities with good healthcare indicators; (m) India has internationally certified doctors (FS); (n) India has internationally accredited hospitals/medical facilities (FS) and India has internationally educated doctors (FS); (o) India is an exotic tourist destination (TD).

### Satisfaction with medical tourism

Hierarchical regression analysis (Table 4) showed that when included facility and services factor with the other three predictors in step 4, country environment factor (β = 0.15, *p* = 0.007), tourism destination factor (β = 0.16, *p* = 0.002), medical tourism costs factor (β = 0.16, *p* = 0.001) and facility and services factor (β = 0.24, *p* < 0.001) contributed significantly to the regression model (*F* = 39.34, *p* < 0.001) and accounted for 28% variations in in outcome variable.

**Table 4:** Linear regression depicting factors influencing study participants' satisfaction toward medical tourism in India.

**Variables**	***R^2^***	**Δ*R^2^***	***B***	***SE***	**β**	***t***	***p***
**Step 1**	0.16	0.16					
Country environment			7.21	0.84	0.40	8.58	< 0.001
**Step 2**	0.20	0.04					
Country environment			5.26	0.93	0.29	5.63	< 0.001
Tourism destination			4.04	0.92	0.23	4.41	< 0.001
**Step 3**	0.23	0.04					
Country environment			3.78	0.98	0.21	3.86	< 0.001
Tourism destination			3.65	0.90	0.21	4.05	< 0.001
Medical tourism cost			3.41	0.82	0.21	4.17	< 0.001
**Step 4**	0.27	0.04					
Country environment			2.65	0.98	0.15	2.69	0.007
Tourism destination			2.79	0.90	0.16	3.11	0.002
Medical tourism cost			2.64	0.81	0.16	3.24	0.001
Facility and services			5.22	1.11	0.24	4.71	< 0.001

Step 1: *R*^2^ = 0.16, (*F* = 73.57, *p* < 0.001); Step 2: Δ*R*^2^ = 0.04, (*F* = 48.24, *p* < 0.001); Step 3: Δ*R*^2^ = 0.04, (*F* = 39.34, *p* < 0.001); Step 4: Δ*R*^2^ = 0.04, (*F* = 36.69, *p* < 0.001).

## Discussion

This study found that more than three-fourths of the study participants traveled to India for treatment purposes. There are around 21,000 government doctors and 93,358 registered physicians in Bangladesh. The population per registered physician is 1,581 (56). Previous literature (24, 57) highlights that there is a long line of sufferers as a result. People line up like bees in Upazila Health Complexes (UHC) and government hospitals to receive medical care because 20 percent of the country's population still lives in poverty, and 80 percent reside in rural areas. These facilities offer services for nearly no cost. Unfortunately, communication with these impoverished individuals is frequently ineffective, and their issues are rarely correctly handled. Sometimes counsel and therapy are given without providing enough information, and patients' illnesses are left undiagnosed, leading to side effects and other difficulties, even causing death. Consequently, a large cohort of people lost their faith and dependability on doctors.

Many Bangladeshi patients travel to India not just to receive complicated procedures but also for routine pathological tests and to confirm diagnoses. Our study reported that 11% of the study participants traveled to India to diagnose their health problems which is a prerequisite to providing appropriate treatment and medication. Patients often mention that although some medical technologies are available in Bangladesh, they have lost faith in doctors and their use of these technologies. In Bangladesh, doctors often cannot identify the actual health problem of the patients as they spend inadequate time during the appointment. Consequently, the patients cannot effectively participate in the medical consultation to express their anxiety and describe their background history, which might be helpful for the doctors to diagnose the disease of the patients more accurately. Also, the doctors earn a sizeable portion of the money that patients pay to that diagnostic center due to their advice to patients to provide samples to that facility. This is why the doctors cannot allow and believe in the test report that is done in other diagnostic centers beyond their preference and suggestions. Interestingly, the accuracy level of some tests depends on the skill and expertise of the health care providers who check the patient's health, such as ultrasound. Therefore, the findings of the test reports often vary due to the difference in the laboratory which makes the patients puzzled and disappointed. This might be an explanation for a number of the study participants visiting an Indian hospital for diagnosis purposes.

The study findings report that among the four sub-factors of the medical tourism index, the facility and services factor appeared as the most influential determinant of Bangladeshi patients' decision-making process regarding medical tourism in India. About two-thirds of the study participants reported that they decided to go to India mainly for its different facilities and services in the healthcare sector, for example, the availability of well-trained, well-experienced, and famous doctors, high-quality healthcare, hospital/medical facilities with high standards doctors, quality treatments and medical materials, friendly behavior of staff and doctors, and favorable medical tourism image. Indeed, Hospitals in India emphasize their highly skilled practitioners and the use of advanced medical technology, procedures, and facilities as the primary draw for international patients (58).

However, our findings are also in line with previous studies, suggesting that the low cost of treatment and check-ups, insurance coverage restrictions, accessibility to complex medical procedures, ability to receive treatment more quickly, privacy concerns, employer and insurance company endorsements, and higher quality care and medical service providers are frequent reasons given for choosing medical tourism (5–9, 15). In particular, Wongkit and McKercher (9) argued that quality issues were perceived as more significant factors influencing the decision-making process for medical tourism than the cost of treatment, insurance coverage, or privacy concerns. They also argued that the quality of doctors and medical facilities, the required treatment, and quality issues were perceived as more significant factors (9).

According to our study findings, the availability of many cultural or natural attractions/sites and attractive tourist destinations in India was also reported as the influencing factor for deciding on medical tourism in India. Regardless of the medical amenity, India is also a preferred choice of destination for patients from developing countries as well as across border neighboring countries also due to its similar climate, English language, familiarity with the culture, history, food, local language, exotic destination, and international and government accreditation (57).

## Conclusion

We found that facility and services are among our models' strongest predictors. Our research demonstrates that the availability of well-trained, well-experienced, and famous doctors, high-quality healthcare, hospital/medical facilities with high standards doctors, quality treatments and medical materials, friendly behavior of staff and doctors, and favorable medical tourism image are essential influencing items of facility and services factor. On the contrary, while medical tourism has significant benefits, the drawbacks are worth noting. Among the items relating to the medical tourism index, the airfare, treatment cost, weather, language, and cultural similarity scored lower, implying disagreement toward these items.

This research has some managerial and theoretical implications. It provides insights into why patients from Bangladesh travel across the border to India for medical treatment. The findings of this research made several contributions to the literature on cross-border medical tourism. Our study provides an empirical and practical basis for medical tourism. Government institutions may tie up with high-quality hospitals in renowned medical tourism destinations in India to ensure public health and safety. Accordingly, this study suggests Indian government widen access to the right food, lessen the language barrier, reduce the airfare for medical tourists, and make the treatment cost more affordable for patients from South Asia and other less developed countries.

Notably, the theory of planned behavior offered some clues for explaining how a person's attitude and subjective norm can influence their intention toward medical tourism. The findings illustrate the importance of the theory of planned behavior as a comprehensive heuristic framework for Bangladeshi people's decision-making process of medical tourism in India.

The study has several limitations. First, the Chittagong city-based study covers the visitors who came to apply for or take the medical visa at the Indian visa center Chittagong (IVAC) during the study period. Covering more administrative divisions could have made the research more vigorous. Second, it is difficult to determine the causality since the study was designed as cross-sectional. Third, collecting data in a short period may have influenced the participants' information. Seasonality could have an impact on the validity of the findings. Finally, an important limitation of the study is that it was conducted with a convenience sample of study participants.

Larger and broader samples could be addressed in future research. A prospective study should include push factors of medical tourism in India so that the government of Bangladesh can address these factors. To expand the overall knowledge base, Bangladeshi tourists' decision-making process to choose and travel to various medical tourism destinations, including India, should be planned as a comprehensive study. Future studies could use qualitative methods to know deep insight into the topic.

## Data availability statement

The raw data supporting the conclusions of this article will be made available by the authors, without undue reservation.

## Ethics statement

Ethical approval was taken from the Institutional Review Committee of the University of Chittagong (CU-SOC-21-0008) as the study involves interviewing human subjects. Written informed consent for participation was not required for this study in accordance with the national legislation and the institutional requirements.

## Author contributions

Investigation: MZ, MAI, AB, NP, and JX. Data analysis: MZ and MKI. Original draft preparation: MZ and JX. Conceptualization, review, and editing: MAI, AB, NP, MKI, FC, and JX. Supervision: FC. All authors have read and agreed to the published version of the manuscript.
